# Antimicrobial Peptides Versus Antibiotics in Farm Animal Production

**DOI:** 10.3390/antibiotics14111108

**Published:** 2025-11-03

**Authors:** Boris Gavrilov, Slavena Davidova, Anastasiia Generalova, Alexandra Gergova, Galina Satchanska

**Affiliations:** 1 Huvepharma EOOD, Biologics Development, Nikolay Haytov Str. 3A, 1113 Sofia, Bulgaria; boris.gavrilov@huvepharma.com; 2 UPIZ Educational and Research Laboratory of Biology—MF, New Bulgarian University, Montevideo Blvd. 21, 1618 Sofia, Bulgaria; stdavidova@nbu.bg (S.D.); f116172@students.nbu.bg (A.G.); 3 UPIZ Virology—BF, New Bulgarian University, Montevideo Blvd. 21, 1618 Sofia, Bulgaria; f104819@students.nbu.bg; 4 Department of Natural Sciences, New Bulgarian University, Montevideo Blvd. 21, 1618 Sofia, Bulgaria

**Keywords:** antimicrobial peptides, antibiotic resistance, livestock production, antimicrobial mechanisms, nano-delivery systems, sustainable agriculture

## Abstract

The increasing prevalence of antimicrobial resistance in livestock pathogens necessitates the development of effective alternatives to conventional antibiotics. This review aims to assess the potential of antimicrobial peptides (AMPs) as alternatives to traditional antibiotics in farm animal production systems, examining their mechanisms of action, efficacy, and applications. A thorough examination of recent literature was conducted to evaluate the properties, classification, and mechanisms of action of AMPs, their natural occurrence, and their applications in poultry, swine, and ruminant production. The review also compared AMPs with conventional antibiotics, antifungals, and antiparasitic drugs. Specific AMPs have shown effectiveness against livestock pathogens, including *Escherichia coli*, *Salmonella*, and *Clostridium perfringens*, along with drug-resistant fungi. One of the primary benefits of AMPs is their strong antimicrobial activity against a wide range of pathogens relevant to farm animal health. Innovative delivery systems, such as self-assembly techniques and nanoparticle encapsulation, can tackle the stability and bioavailability issues associated with AMP administration in farm settings. AMPs represent promising alternatives to conventional antimicrobials in livestock production, offering significant benefits, including a reduced risk of resistance development, immunomodulatory effects, and broad-spectrum activity. However, addressing disadvantages related to production costs, stability, and delivery systems is crucial for their successful commercial application. Integrating AMPs into sustainable farming, after overcoming their shortcomings, could significantly contribute to global efforts to combat antimicrobial resistance.

## 1. Introduction

**Figure 1:**
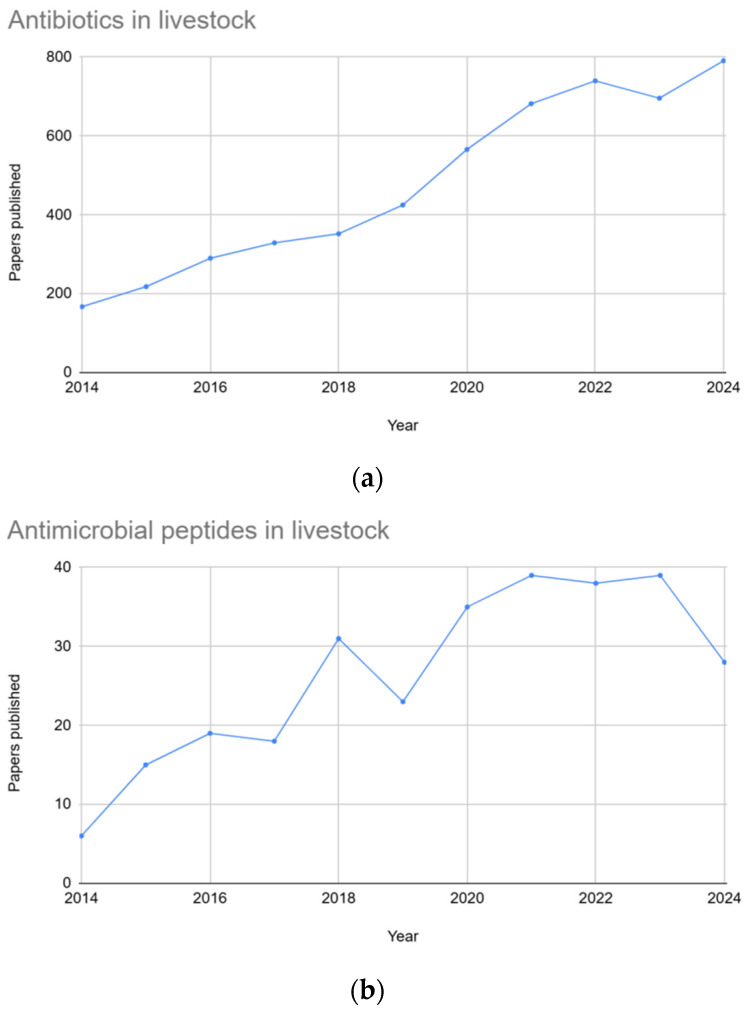
Graphs showing the number of publications over a ten-year period (2014–2024). Graph (**a**) depicts the number of papers published using the keywords “antibiotics in livestock”, whereas graph (**b**) depicts the number of papers published using the keywords “antimicrobial peptides in livestock”.

The agricultural industry heavily relies on antimicrobials for disease management and enhancing productivity in livestock, including aquaculture, poultry, cattle, and pigs [1,2,3]. However, rising concerns regarding antimicrobial resistance (AMR) and potential toxicological safety problems necessitate exploration of alternative strategies [2,4,5]. The growing concern regarding antimicrobial resistance in foodborne pathogens further highlights the importance of developing effective alternatives to antibiotics. Between 2014 and 2024, there has been a steady increase in publications on both topics, with significantly higher interest in antibiotics for livestock, while the growing attention to antimicrobial peptides reflects a rising interest in antibiotic alternatives (Figure 1).

In 1997, the WHO determined that “antimicrobial overuse leads to the selection of resistant forms of bacteria in the ecosystem of use” and recommended that if an antibiotic was essential to human treatments, it should not be used as a growth promoter in animals. This is because after antibiotic administration, some residues can remain in animal products, leading to the development of resistance [6]. In 2006, the European Union prohibited the use of antibiotics as growth promoters. In 2015, the “Global Action Plan to Combat Antimicrobial Resistance” was launched through a partnership between the FAO and the WHO, with the aim of maintaining the effectiveness and safety of infectious disease treatment.

**Figure 2:**
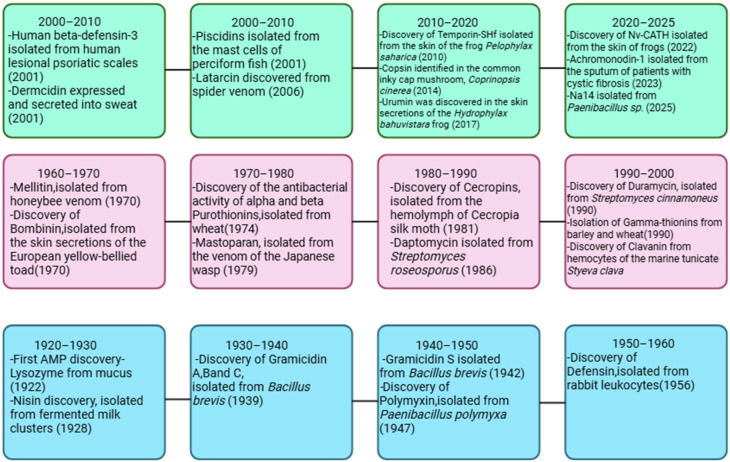
Timeline of key milestones in the discovery of AMPs (1922–2025). Figure made with Biorender (https://www.biorender.com/, accessed on 3 October 2025).

Following this, announcement No. 194 by the Ministry of Agriculture and Rural Affairs of the People’s Republic of China established a management policy that mandates the cessation of veterinary growth promotion drugs by 2020. Consequently, there is an urgent need for innovative substances with strong biological activity that can serve as alternatives to antibiotics in animal husbandry. Currently, several categories of agents have been proposed as in-feed replacements for antibiotics, including lysozymes, glucose oxidases, organic acids, phytogenic essential oils, bacteriophages, and AMPs. Additionally, prebiotics, probiotics, and synbiotics have received considerable attention for their potential to alter the intestinal microbiota of animals, thus promoting gut health and enhancing disease resistance. Among these promising substances, AMPs are notable for their broad-spectrum antimicrobial properties and diverse biological functions, which include promoting growth in animals, influencing gut microbiota, modifying gut structure, and enhancing disease resistance. Therefore, they hold considerable promise for application in various fields, especially as alternatives to in-feed antibiotics [7,8]. On Figure 2 is presented a timeline of key milestones in the discovery of AMPs.

Carbapenemase-producing *Enterobacteriaceae* (CPE), historically regarded as a human-associated threat, have now also been identified in farm animals [9]. Recent surveillance data indicate that CPE have been detected in 14 out of the 30 European Union member states—equivalent to nearly 50%—a trend that is particularly alarming in the context of zoonotic transmission and food chain contamination [9].

**Figure 3:**
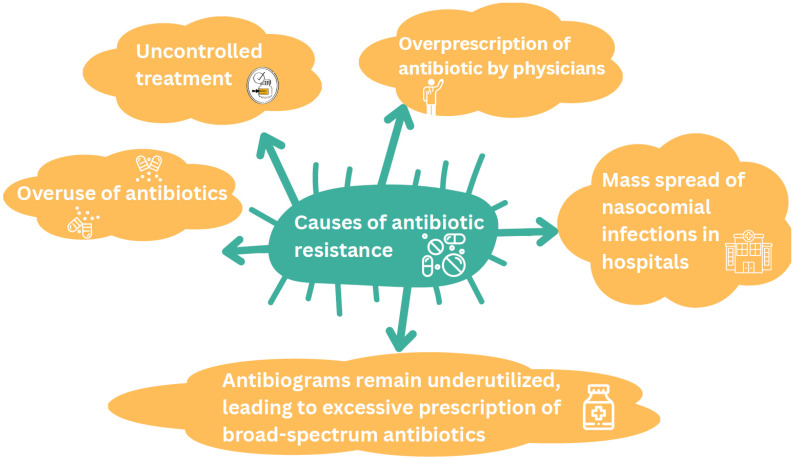
Diagram depicting the leading causes of antibiotic resistance.

The scale of antimicrobial resistance is alarming, with an estimated 500,000 people infected worldwide. According to projections by O’Neill and colleagues [10], antimicrobial resistance is expected to cause approximately 10 million deaths by 2050 [11]. This global challenge has prompted governments and public health agencies to increase efforts in antimicrobial resistance surveillance and research. Bacteria, including *Pseudomonas aeruginosa*, *Staphylococcus aureus*, coagulase-negative *Staphylococcus*, *Salmonella* spp., *Shigella* spp., *Enterococcus* spp., and *Escherichia coli*, currently demonstrate the highest levels of antibiotic resistance and cause the most serious infections in both humans and animals [4]. Of particular concern is the potential transfer of antibiotic resistance from intestinal bacteria in animals to humans through consumption of foods of animal origin (Figure 3) [4,12].

Recent estimates suggest that global antimicrobial use in livestock could reach approximately 143,481 tons by 2040 under business-as-usual scenarios, representing a 29.5% increase from baseline levels in 2019 [13]. This upward trend underscores the pressing need for effective alternatives, particularly in light of the global commitments outlined in the Muscat Manifesto to reduce antimicrobial use in agrifood systems by 30–50% by 2030 [13]. Using an improved methodology that considers animal weight at treatment rather than at slaughter, researchers have estimated that global antimicrobial use in cattle, chickens, and pigs amounts to 76,060 tonnes annually, representing a more accurate assessment of the scale of the AMR challenge [1]. Antimicrobial peptides (AMPs), also referred to as host defense peptides (HDPs), have emerged as one prospective class of alternatives for use in livestock production systems, including aquaculture, poultry, ruminants, and swine [14,15,16]. AMPs are naturally occurring short chains of amino acids (approximately 10–100 residues long and higher, Table 1) that are critical parts of the innate immune system of diverse organisms and are ubiquitous in nature. They are usually cationic with amphipathic properties, enabling them to interact with microbial membrane and cell wall and making them potent against a broad spectrum of pathogens [17]. 

This review explores the potential applications, advantages, and limitations of AMPs as alternatives to conventional antibiotics in livestock production, with a particular focus on their antimicrobial efficacy, mechanisms of action, and challenges to widespread implementation.

## 2. AMPs Against Bacteria, Fungi, and Parasites in Farm Animal Production

AMPs have emerged as potential alternatives to conventional antibiotics in aquaculture (Table 2) and farm animal production due to their broad-spectrum antimicrobial activities and immunomodulatory functions. The growing concern over antibiotic resistance has accelerated interest in AMPs as potential substitutes to improve growth performance, intestinal health, and immunity in livestock species [14].

### 2.1. AMPs Against Bacterial Infections in Farm Animals

#### 2.1.1. Application of AMPs in Poultry

The application of antimicrobial peptides in combating poultry pathogens is of particular interest due to their broad spectrum of activity, low likelihood of resistance development, and multifunctional immunomodulatory properties.

AMPs have demonstrated significant benefits in controlling necrotic enteritis (NE) caused by Clostridium perfringens, which has become a significant economic concern, especially in countries that have banned antibiotic growth promoters [25]. Researchers have identified enterococci strains with antimicrobial activity against *C. perfringens* that could serve as protective strains in poultry farming. For instance, *E. faecium* X2893 and X2906 strains, which produce enterocin A and enterocin B (Table 3), have demonstrated significant inhibitory effects against *C. perfringens* without carrying acquired resistance genes, making them compelling candidates for protective strains [25].

Peptides affecting the outer membrane lipid asymmetry system (MlaA-OmpC/F) in avian pathogenic *Escherichia coli* (APEC) have shown significant potential for reducing colonization in chickens. Three peptides derived from *Lactobacillus rhamnosus* GG (Table 3) have demonstrated inhibitory activity against APEC and effectively reduced cecum colonization in chickens. These peptides work by disrupting the APEC membrane and cell wall, either by causing membrane shedding, rupturing, or flaccidity, and downregulating the expression of *ompC*, *ompF*, and *mlaA* genes responsible for maintaining outer membrane lipid asymmetry [33].

Several avian AMPs have been evaluated for their efficacy in poultry production. Truncated cathelicidins CATH-1(6–26) (Table 3) and CATH-2(1–15), along with avian β-defensins (including ABD1, ABD2, ABD6, and ABD9) (Table 3), have shown potent antibacterial activities against important poultry pathogens such as *E. coli*, *Salmonella* Enteritidis, *Salmonella* Typhimurium, *Campylobacter jejuni*, and *Clostridium perfringens* [27]. Notably, in ovo administration of ABD1 significantly reduced early chick mortality (by 44%) from experimental yolk sac infection caused by avian pathogenic *E. coli*, demonstrating protection comparable to CpG ODN, a known immune stimulant [27].

Studies investigating the efficacy of AMPs across various production contexts highlight their multifaceted benefits beyond direct antimicrobial activity. For instance, HDP-cLF36, derived from camel lactoferrin, increased *Lactobacillus* spp. and decreased harmful bacteria in the ileum of *E. coli*-contaminated chickens, while upregulating genes associated with immune cells and tight junction proteins [14]. Similarly, sublancin reduced *C. perfringens* counts in the cecum of contaminated chickens, improved villus height and villus height to crypt depth ratio in the duodenum, and decreased pro-inflammatory cytokine levels in the ileum [14].

#### 2.1.2. Application of AMPs in Swine Production

In swine production, various AMPs have been investigated to combat post-weaning diarrhea, a serious problem caused primarily by enterotoxigenic *E. coli* (ETEC). Dietary supplementation with AMPs, such as microcin J25, cecropin AD (Table 4), and composite AMPs containing lactoferrin, cecropin, a member of the defensin class, and plectasin, has effectively reduced diarrhea incidence and improved growth performance in weaned piglets [14]. For example, cecropin AD supplementation significantly reduced diarrhea incidence by 47.6% and improved intestinal morphology, nitrogen retention, and dietary energy digestibility in ETEC-contaminated piglets [14].

**Figure 4:**
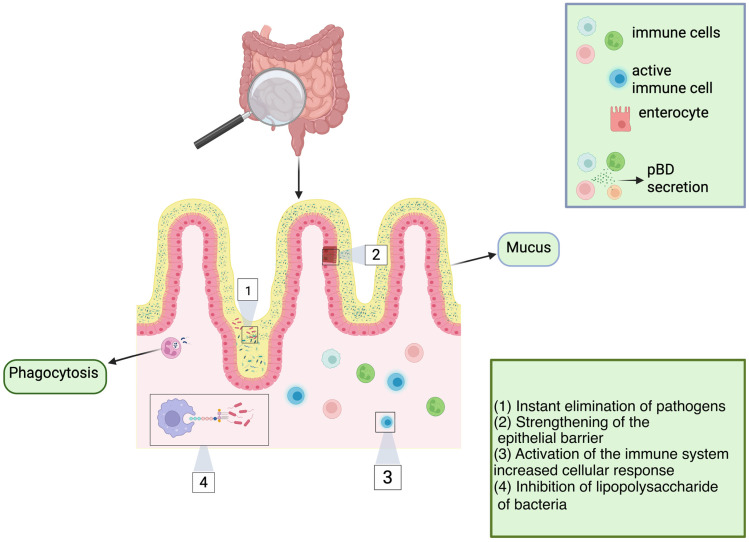
Multifunctional roles of pBD. Porcine β-defensins (pBDs) are secreted by a variety of specialized cells of the immune system. The roles of pBDs extend beyond direct antimicrobial activity against bacteria, viruses, and fungi; pBDs also possess the ability to neutralize lipopolysaccharides (LPS). These findings suggest that pBDs play a pivotal role in immunomodulating host cells, triggering immune activation and boosting cellular immune responses, thereby enhancing the ability of the host to combat target pathogens. Some of these mechanisms include enhanced phagocytosis, fine-tuned activation of the immune system, and the strengthening and maintenance of epithelial barrier integrity. Figure made with Biorender.

One of the most prospective classes of AMPs in agricultural production is β-defensins. Porcine β-defensins (pBDs) (Table 4, Figure 4) are positively charged peptides typically linked to the innate immune response. These molecules possess both antimicrobial and immunomodulatory properties, as evidenced by various trials [15]. The expression of pBD is thought to be regulated by a complex network of factors, including cytokine signaling, hormonal influences, dietary components like Zn^2+^, and the precise control of gene activity via epigenetic modifications [15].

#### 2.1.3. Application of AMPs in Ruminants

AMPs have shown potential in combating many diseases. Some of which are bovine mastitis and respiratory diseases. Peptide H18R (H2) has demonstrated greater efficacy than vancomycin in controlling *Staphylococcus aureus*, which causes mastitis, by being internalized into MAC-T cells and inhibiting MRSA [14]. Similarly, bovine NK-lysin-derived peptides can damage the cell wall and plasma membrane, killing *Mycoplasma bovis*, an important contributor to the bovine respiratory disease complex [14]. In ruminants, AMPs, particularly bacteriocins (Table 5), such as bovicin HC5 and nisin, have shown potential to reduce methane production, which accounts for 2–12% of the gross energy loss from feeds and contributes significantly to agricultural greenhouse gas emissions [14].

### 2.2. AMPs Against Fungal Infections in Farm Animals

Fungal infections present a significant challenge in farm animal production. The currently available antifungal agents are limited, and their widespread use has led to the emergence of antifungal resistance in *Candida*, *Cryptococcus*, and *Aspergillus* species. Therefore, the development of novel antifungals is urgently needed [48].

Antimicrobial peptides with antifungal activity, specifically referred to as antifungal peptides (AFPs) (Table 6), are currently regarded as the most prospective alternative to conventional antifungal agents due to the fact that they are highly selective and less prone to facilitate the selection of drug resistance [48].

**Figure 5:**
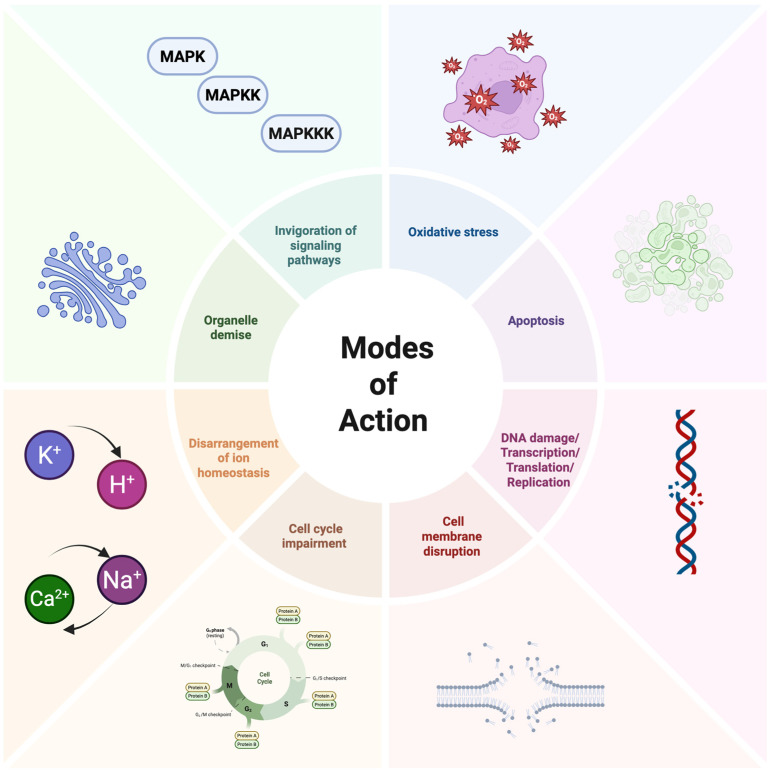
Schematic representation of the mechanisms of action of AFPs. Figure made with Biorender.

The exploration of AFPs’ modes of action against fungi reveals a variety of mechanisms (Figure 5) [51,52]. In a study by Gong et al., the fish-sourced peptide AP10W was shown to exert its fungicidal activity through modes of combined actions, including interaction with the fungal cell walls via laminarin, mannan, and chitin, enhancement of cell wall permeabilization, induction of membrane depolarization, and increase in intracellular reactive oxygen species (ROS) generation [51].

The application of AFPs in poultry production has shown particular potential for controlling fungal diseases such as aspergillosis. Recent studies have demonstrated that certain AFPs isolated from entomopathogenic bacteria show significant activity against important fungal pathogens. These AFPs typically affect cell wall and membrane integrity, producing lethal pores in microorganisms [48].

The application of novel AFPs in food preservation has also shown efficient results against fungal contamination. A study demonstrated that a novel designed membrane-active peptide, A11 (Table 3), modified from acidocin J1132β, exhibits potent inhibitory activity against fungal pathogens, as well as a favorable safety profile [26]. A11 caused transient membrane permeabilization and killed fungal cells through membrane depolarization and/or intracellular interactions with fungal DNA, while maintaining most of its inhibitory effects even when heated to temperatures up to 100 °C [26].

Notably, certain AFPs or their derivatives may also have potential as feed additives in livestock production to prevent fungal contamination. Some studies have found that autoclaved cultures containing thermostable antimicrobial compounds may not be harmful when added as food supplements, while retaining their antimicrobial activity against fungi [53].

This suggests the possibility of developing AFP-based feed additives that could help prevent fungal infections in farm animals without the need for therapeutic intervention.

### 2.3. AMPs Against Parasitic Infections in Farm Animals

Parasitic infections, including those caused by protozoan pathogens such as *Leishmania* and *Histomonas*, are becoming increasingly resistant, posing substantial challenges to animal health and welfare in agricultural settings [53,54]. Traditional treatment approaches have relied heavily on chemical antiparasitic drugs, but the emergence of resistance has necessitated the exploration of novel therapeutic strategies [53]. In this context, AMPs derived from various sources, including insects, offer unique advantages as potential antiparasitic agents.

The entomopathogenic bacteria of the genera *Xenorhabdus* (Table 7) and *Photorhabdus* have emerged as particularly valuable sources of antimicrobial peptides with potential antiparasitic activity. These bacteria, which form symbiotic relationships with entomopathogenic nematodes, produce a variety of non-ribosomal templated peptides (NR-AMPs) that exhibit broad-spectrum antimicrobial properties [53]. Importantly, studies on cell-free culture media (CFCM) from *Xenorhabdus budapestensis* and *X. szentirmaii* have demonstrated potent activity against both the promastigote and amastigote forms of *Leishmania*, as well as against *Histomonas meleagridis*, which causes histomonosis (blackhead disease) in poultry [52]. This ability to target different life stages of parasites is particularly valuable for developing effective control strategies in livestock production.

**Figure 6:**
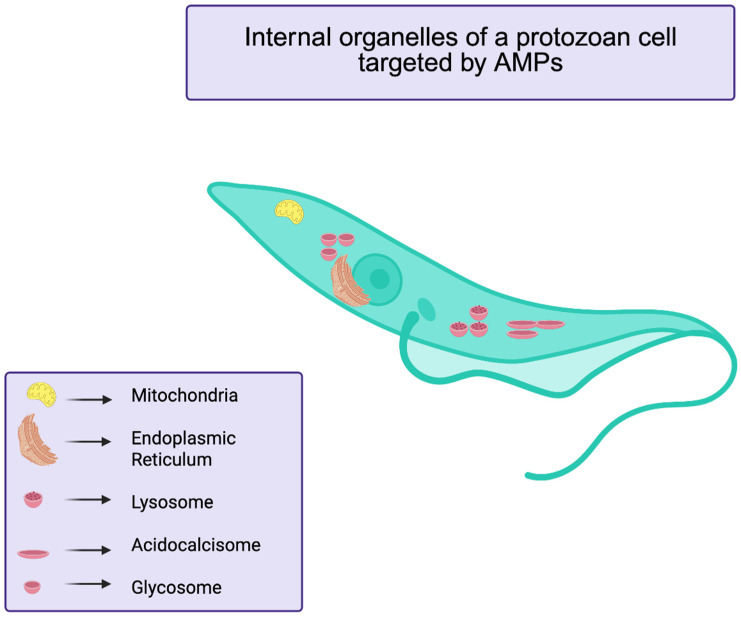
Schematic overview of a protozoan cell with various internal targets of AMPs. Figure made with Biorender.

The mechanism of action of peptides against parasites appears to be multifaceted (Figure 6). For instance, research on Halictine-2, a dodecameric AMP isolated from the venom of the eusocial honeybee *Halictus sexcinctus*, has shown that its variant P5T demonstrates significant leishmanicidal activity through membrane disruption and induction of apoptosis-like cell death [58]. Electron microscopy analyses revealed distinct pores on the parasite membrane after exposure to this peptide, suggesting a direct membrane-disruptive mechanism of action. Additionally, biochemical investigations indicated that treatment with this AMP led to increased ROS production, calcium efflux, mitochondrial depolarization, and ATP depletion in the parasites, culminating in programmed cell death [58,59].

In contrast to most AMPs from higher eukaryotes that usually work in the micromolar range, certain cyanobacterial antiparasitic peptides are active at picomolar concentrations [60]. This remarkable potency demonstrates the diversity of action mechanisms that various AMPs can employ against parasitic targets. Whereas AMPs from higher eukaryotes typically disrupt the plasma membrane, cyanobacterial peptides often target intracellular processes, providing additional strategies for antiparasitic action.

Another encouraging aspect of AMPs for farm animal applications against parasites is their potential for combinatorial therapy with existing antiparasitic drugs. Research has demonstrated enhanced killing efficacy when certain AMPs are co-administered with conventional antiparasitic agents. For instance, the combination of P5T with potassium antimony tartrate (PAT) showed synergistic effects against *Leishmania* parasites [58]. Such combinatorial approaches could help address problems of drug resistance while potentially allowing for reduced dosages of conventional drugs, thereby minimizing residues in animal products.

A key advantage of AMPs in farm animal production is their potential to have a minimal impact on host cells. For example, the aforementioned Halictine-2 variant P5T exhibited negligible hemolytic activity against human erythrocytes and low cytotoxicity toward mouse macrophages, suggesting a favorable safety profile [58]. This selectivity is crucial for developing antiparasitic treatments that can be safely administered to livestock without detrimental effects on animal health or product quality.

### 2.4. Synthetic AMPs in Farm Animals

There is growing research into synthetic AMPs formulated for animal feed to improve livestock health and reduce antibiotic use, though specific commercial products are still emerging [61].

Cathelicidins are a group of AMPs, including synthetic derivatives such as Bac2A and IDR-1018, derived from bovine cathelicidins. They have demonstrated antibacterial activity and immunomodulatory effects, making them candidates for replacing antibiotics in animal feed and treatment [61,62].

Synthesized AMPs, such as protegrin-1 (PG-1), have shown remarkable efficacy in veterinary applications, with transgenic mice expressing PG-1 exhibiting enhanced resistance against *Actinobacillus suis* infection compared to wild-type counterparts [63]. Other notable synthetic AMPs include BMAP-27 and BMAP-28, which are derived from bovine myeloid cells and effectively inhibit multidrug-resistant bacterial strains in livestock, particularly against pathogens such as methicillin-resistant *Staphylococcus aureus* [64] and pan-drug-resistant *Acinetobacter baumannii* [65].

In poultry production, synthetic AMPs such as SGAMP derived from porcine intestines have demonstrated higher feed efficiency in broilers under conditions of chronic heat stress [66], while also showing positive effects against infectious bronchitis virus in chickens [67]. These synthetic peptides are increasingly being incorporated into animal feed formulations as alternatives to antibiotic growth promoters, helping to maintain gut health, boost immunity, and prevent bacterial inflammation without contributing to antimicrobial resistance [68].

Ultimately, antimicrobial peptides offer multifaceted benefits in farm animal production as alternatives to conventional antimicrobial drugs for controlling bacterial, fungal, and parasitic infections. The diverse mechanisms of action of AMPs, their selectivity toward microbial targets, and limited propensity to induce resistance make them attractive alternatives to conventional antimicrobial drugs. Their efficacy against a wide range of pathogens, along with their immunomodulatory functions, contributes to improved animal health, productivity, and food safety.

## 3. Antibiotics, Antifungals, and Antiparasitic Drugs in Farm Animal Production

The widespread administration of antibiotics, antifungals, and antiparasitic drugs to farm animals serves multiple purposes: treating infections, preventing diseases, and in some countries, promoting growth. However, these practices have increasingly raised concerns about their contribution to global antimicrobial resistance (AMR) and other public health challenges [69].

### 3.1. Antibiotics in Farm Animal Production

**Figure 7:**
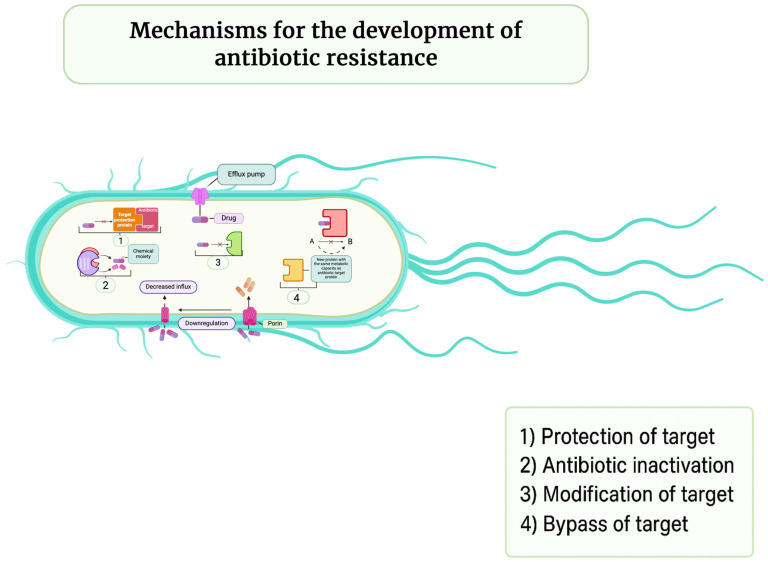
Mechanisms for the development of antibiotic resistance. (1) Bacteria can produce proteins that protect the antibiotic’s target without directly altering it. These protective proteins bind to the target (often ribosomes or enzymes) and prevent the antibiotic from interacting with it, while still allowing the target to perform its normal function. (2) Antibiotic inactivation is one of the most common resistance mechanisms. Bacteria generate enzymes that chemically inactivate the antibiotic, rendering it ineffective before it reaches its target. (3) They also modify the structure of the molecule (usually a protein or enzyme) to which the antibiotic binds. This change reduces or eliminates the antibiotic’s ability to bind and inhibit the target. (4) Additionally, bacteria can develop alternative metabolic pathways that bypass the step blocked by the antibiotic. Even with the antibiotic present, the bacteria find another way to perform the same vital function. “A” represents the original antibiotic target while “B” represents a newly acquired or modified enzyme or pathway that performs the same essential function as “A” but is not inhibited by the antibiotic. Figure made with Biorender.

The agricultural industry utilizes approximately 70% of all globally produced antibiotics, creating selective pressure for resistant bacterial strain development that can be transmitted to humans through direct contact, contaminated food, or environmental exposure [28,44]. Bacterial resistance mechanisms include enzymatic degradation, target modification, reduced membrane permeability, efflux pump activation, and horizontal gene transfer, which accelerates the spread of resistance beyond vertical transmission (Figure 7) [44]. Regulatory frameworks have emerged in response, with the EU banning antibiotic growth promoters in 2006 and the US introducing the Veterinary Feed Directive in 2017; however, global consumption continues to rise in emerging economies with less stringent regulations [28].

Common agricultural antibiotics include β-lactams, tetracyclines, macrolides, and fluoroquinolones, many of which are also critical for human medicine, raising concerns about cross-resistance [28]. Specific pathogens demonstrate concerning resistance patterns, such as *Salmonella Infantis* in broiler flocks (3.7% flock-level prevalence in the Netherlands) carrying pESI-like mega-plasmids with genes for antibiotic resistance, virulence, and fitness, showing resistance to aminoglycosides, sulphonamide, tetracycline (93%), trimethoprim (71%), and fluoroquinolones [70]. Following Korea’s 2011 ban on antimicrobial growth promoters, *Clostridium perfringens* isolates showed increased resistance to multiple antimicrobials, with significantly higher resistance to gentamicin, clindamycin, and virginiamycin in isolates from chickens with necrotic enteritis compared to those from healthy chickens [71].

Antimicrobial agents can have a significant impact on the animal gut microbiota. Antibiotics like enrofloxacin or doxycycline administered to APEC-infected turkeys altered cecal microbiota metabolic activity and disrupted short-chain fatty acid production [72]. Conversely, studies have demonstrated that authorised salinomycin treatment enriches *Bacteroidetes* in the cecal microbiota of broilers, whereas vaccinated birds exhibit higher abundances of *Firmicutes* and *Proteobacteria*. Notably, the enrichment of *Bacteroidetes* correlates with improved growth performance in salinomycin-supplemented birds [73,74,75]. Similarly, *Ferulago angulata* extract (400 mg/kg) positively influenced immune status, growth performance, and intestinal microbiota in *Campylobacter*-infected broilers, reducing *C. jejuni* and coliform populations with performance comparable to salinomycin [76]. Additionally, recent research has identified salinomycin as a promising antiviral compound that inhibits porcine epidemic diarrhea virus (PEDV) replication in Vero cells in a dose-dependent manner [77].

Antibiotic growth promoters (AGPs) such as flavophospholipol and virginiamycin are known to induce dynamic microbial shifts in broiler chickens, potentially enhancing anti-inflammatory pathways and nutrient absorption. However, virginiamycin has also been associated with increased colonization by potentially pathogenic bacteria, including *Clostridium perfringens*, *Campylobacter*, and* Escherichia*/*Shigella* species [78]. Among these, *Campylobacter* poses a notable public health risk. Studies have shown that dairy cattle can act as reservoirs for *C. jejuni* strains genetically similar to those infecting humans. Additionally, beef cattle farms have been found to harbor antibiotic-resistant *Campylobacter* isolates [79]. In a related shift in our understanding of ruminal microbiota, recent research has demonstrated that standard enrichment methods intended to isolate *Fusobacterium necrophorum* favor the growth of *F. varium* instead. This finding suggests a potential paradigm shift in our understanding of the rumen ecosystem and its implications for cattle health [80].

Exposure to monensin can select for resistant *Staphylococcus aureus* mutants that exhibit increased growth rates and virulence, with resistance associated with the derepression of de novo purine synthesis through mutations in transcriptional regulators, such as purR [81]. When broilers were infected with coccidiosis, researchers observed microbiota shifts with increases in *Erysipelotrichaceae*, *Lactobacillaceae*, *Bacteroidaceae*, *Streptococcaceae*, and *Peptostreptococcaceae* in both control and monensin groups, while functional oil blend supplementation partially mitigated these variations [82,83].

Flavophospholipol shows application in controlling resistance, with studies demonstrating that 64 ppm supplementation significantly suppressed increases in tetracycline-resistant *E. coli* in pig feces by inhibiting conjugational transfer and growth [84]. Similarly, flavophospholipol (64 ppm) reduced the acquisition of resistance to ampicillin, streptomycin, and tetracycline by recipient *Salmonella* Enteritidis strains in chickens [85]. However, flavophospholipol treatment in *Salmonella* Typhimurium-contaminated pigs increased *Proteobacteria* while decreasing *Firmicutes* and *Roseburia*, suggesting possible dysbiosis, though *Lactobacillus* abundance increased [86].

Alternative approaches include synbiotics, with research showing that *Lactobacillus reuteri*, *Enterococcus faecium*, *Bifidobacterium animalis*, and *Pediococcus acidilactici* culture supernatants decreased *Clostridium perfringens* proliferation in vitro, while in vivo supplementation increased villi height, reduced IL-1 mRNA, increased IL-10 mRNA, lowered cecal *C. perfringens* loads, and increased bile anti-*C. perfringens* IgA [87]. Sophorolipids have shown prospective antimicrobial activity against *Eimeria maxima* and *Clostridium perfringens* while improving gut health in necrotic enteritis-afflicted broilers [35].

Cross-disciplinary research has identified COVID-19 drug repurposing candidates that regulate genes related to cholesterol homeostasis and microtubule cytoskeleton organization (36% of active compounds), potentially offering new approaches to antimicrobial resistance [88].

### 3.2. Antifungals in Farm Animal Production

**Figure 8:**
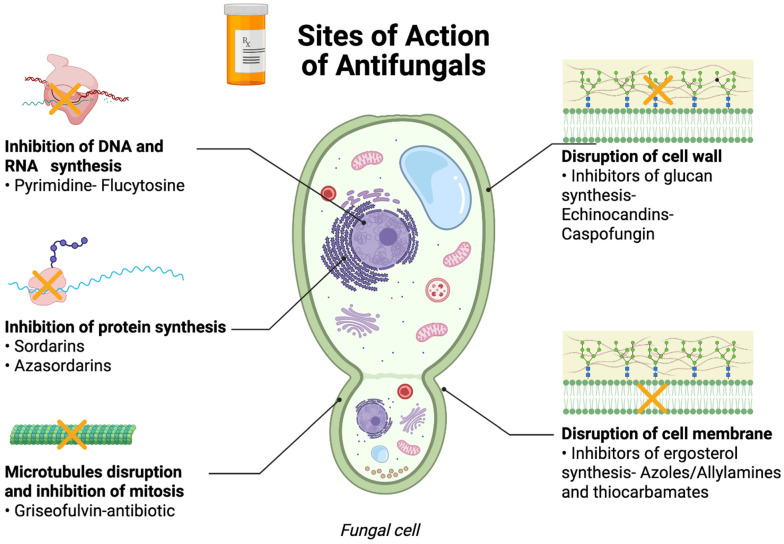
Classes of Antifungal Drugs and Their Overall Mechanism of Action. Figure made with Biorender.

Fungal diseases pose significant threats to human, animal, and food security globally, with limited antifungal options leading to cross-use between agriculture and healthcare, promoting resistance (Figure 8) [89,90,91,92]. Climate change exacerbates these challenges by promoting thermotolerance in fungal pathogens and the emergence of resistant species, while agricultural antifungals, similar to those used in human medicine, contribute to resistance in environmental fungi [91].

Veterinary antifungal use faces numerous challenges, including limited registered products, concerns about the mechanism of action, side effects, and withdrawal periods, with a notable lack of comprehensive surveillance compared to antibiotics [93]. Quantitative data reveal minimal usage in companion animals, with fungicide azoles accounting for 37% of the 2.05 tons of veterinary antifungals sold in France in 2022 [93]. Cross-resistance between agricultural fungicides and medical antifungals presents serious concerns, particularly with triazoles, as exemplified by potential cross-resistance between iprolufenoquin and olorofim [91].

Fungal infections in farm animals range from superficial to systemic, with *Candida tropicalis* emerging as a significant azole-resistant pathogen in dairy cow mastitis [94]. Aspergillosis occurs frequently in companion and zoo animals, but less commonly in livestock. However, poultry farms often have high environmental *Aspergillus* spore loads [93]. A Dutch study found that 11.3% of veterinary *Aspergillus fumigatus* isolates were azole-resistant across various animals, with resistance primarily mediated by cyp51A [95]. *Trichophyton mentagrophytes* in pigs demonstrates concerning zoonotic potential, with documented transmission to farm workers [96].

Treatment approaches include various azoles (itraconazole being most common) and traditional amphotericin (AmB), though the latter often proves ineffective, and azole overuse leads to resistance [93,96,97]. Advancing alternatives include nanotechnology-enabled antifungals, such as metal/metal oxide nanocomposites and nanoemulsions, which exhibit high antimicrobial activity [97]. Novel antifungals with veterinary potential include fosmanogepix for drug-resistant mold infections, olorofim for endemic fungi, and inhaled formulations like opelconazole for respiratory fungal infections [91].

Preventive measures and future directions encompass improved housing, proper hygiene, appropriate stocking densities, environmental disinfection, novel compounds like 1,2,4-triazolyl-α-amino acids and dipeptides, and regular surveillance of resistance patterns [49,95,96].

### 3.3. Antiparasitic Drugs in Farm Animal Production

**Figure 9:**
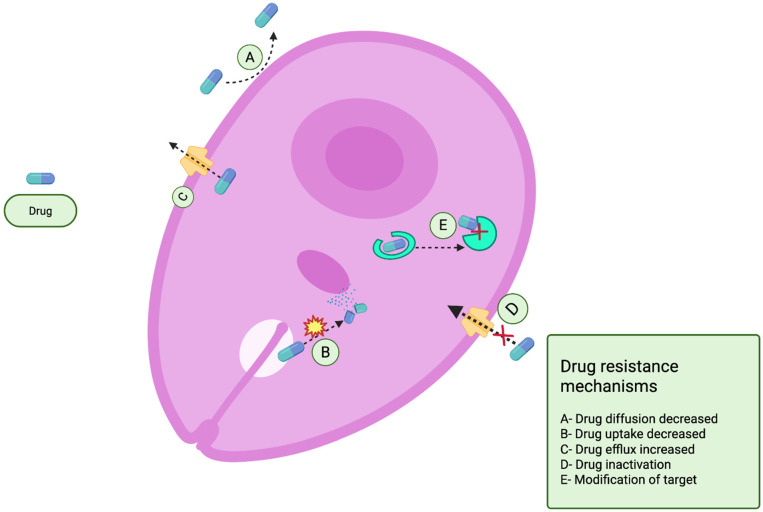
Drug-resistance mechanisms employed by amastigotes of *Leishmania* spp. With letters, different processes are represented. “A”—Decrease of drug diffusion; “B”—Decrease of drug uptake; “C”—Increase of drug efflux; “D”—Inactivation of drug; “E”—Target modification. Figure made with Biorender.

Parasitic infections pose significant challenges to livestock production worldwide, affecting animal health, welfare, and productivity, with the emergence of drug resistance (Figure 9) threatening the sustainability of parasite control strategies [98,99,100]. Major parasitic threats include gastrointestinal nematodes such as *Teladorsagia circumcincta* in sheep and goats, and *Ascaridia galli* in poultry. Notably, *A. galli* has become more prevalent following the implementation of EU welfare regulations (Directive 1999/74/EC), which transitioned from unfurnished cages to more animal-friendly housing systems with litter, thereby increasing parasite exposure [98,99].

*Eimeria tenella* is one of the most virulent species of coccidia affecting poultry, specifically targeting the ceca of chickens and causing hemorrhagic pathologies [101]. This obligate intracellular protozoan parasite is responsible for significant economic losses to the global poultry industry, with an estimated annual cost exceeding 3 billion USD worldwide [101]. As reported by Rogala-Hnatowska et al., salinomycin is a commonly used poultry feed supplement against *Eimeria* spp. in broiler chickens [102]. Alternative control measures, including dietary polyherbal formulations, have shown prospective results in decreasing the impact of eimeriosis on broilers by exerting coccidiostatic effects against *E. tenella* and other *Eimeria* species [103].

The impact of parasitic infections extends beyond terrestrial livestock to aquaculture (Table 2), where *Ichthyophthirius multifiliis* (Ich) causes white spot disease in fish worldwide, resulting in severe economic losses, high morbidity, and mortality rates [104]. Parasitic infections cause significant damage, as seen with *A. galli* larvae penetrating the intestinal mucosa and causing inflammation, reduced nutrient absorption, and potential mortality. Meanwhile, Ich invades the epithelial layer of fish gills and skin, triggering both innate and adaptive immune responses [98,104].

Control of parasitic infections has traditionally relied on anthelmintic drugs, including benzimidazoles (fenbendazole, flubendazole), imidazothiazoles (levamisole, pyrantel), and macrocyclic lactones; however, their efficacy is increasingly threatened by the development of resistance through intensive selection following repeated treatments [98]. The faecal egg count reduction test (FECRT) serves as the primary diagnostic tool for detecting anthelmintic resistance at the farm level, necessitating standardized experimental design and statistically rigorous susceptibility classification methods [105].

Concerning limitations exist in available treatments, with only benzimidazoles approved for poultry in the EU and US, administered via drinking water, which carries risks of underdosing, repeated treatments (up to six times per production cycle), and exposure to uniform selection pressure [98]. Evidence of resistance is mounting, with *T. circumcincta* showing resistance to multiple drug classes, including mutations in the beta-tubulin isotype 1 gene (F200Y and E198L) found in benzimidazole-resistant isolates. Additionally, documented resistance of Ascaridia dissimilis to fenbendazole has been reported in turkeys. At the same time, aquaculture faces challenges related to chemical treatments, which raise concerns about food safety and the environment [98,99,104].

Alternative approaches to combat resistance include plant-derived compounds like Licochalcone A (Lic A), a licorice-derived flavonoid showing potent activity against *Giardia duodenalis* (IC50 27.42 μM, outperforming metronidazole’s 35.05 μM) by inhibiting trophozoite adhesion, causing structural damage, mitigating weight loss in infected animals, reducing intestinal parasite load, and enhancing host antioxidant capacity [106].

Targeted treatment (TT) approaches offer therapeutic potential for reducing anthelmintic resistance by deworming animals based on evidence of infection severity, rather than calendar-based blanket treatments, thereby maintaining a pool of unselected parasites in refugia to minimize the risk of resistance development [98]. TT implementation requires reliable diagnostics, including post-mortem examination, coproscopic analyses, serological tests, and molecular tools such as McMaster, Mini-FLOTAC, digital droplet PCR, and LAMP-LFD assays [98]. Studies using 200 eggs per gram of feces as a treatment threshold showed lower worm burdens, fewer cumulative fecal eggs, higher egg production, better feed conversion, and improved plumage condition compared to conventional approaches [98].

Advanced genomic analyses provide insights into resistance mechanisms and host–parasite interactions, with chromosome-scale genome assemblies revealing genes associated with resistance in *T. circumcincta* and transcriptomic analyses showing upregulation of immunity-related genes in Ich-infected fish [99,104].

Sustainable parasite control requires continued research into novel agents with multiple mechanisms of action, prudent use of existing drugs, targeted treatment approaches, and improved understanding of resistance genetics to address the critical challenges facing livestock and aquaculture production.

## 4. Prospects of Antimicrobial Peptides Compared to Antibiotics in Treating Livestock Diseases

### 4.1. Antimicrobial Peptides as Alternatives to Conventional Antibiotics

Antibiotics have been extensively used as growth promoters in livestock production, substantially reducing production costs, morbidity, and mortality while promoting animal production performance [107]. The widespread emergence of antimicrobial resistance (AMR) presents a significant challenge to both human and animal health, with alarming projections suggesting that AMR could be responsible for approximately 10 million deaths by 2050 if effective countermeasures are not implemented [61]. This silent pandemic has particularly affected the livestock industry, where the excessive and imprudent use of antibiotics has contributed to the rise of multidrug-resistant (MDR) bacterial strains and contamination of animal products with antibiotic residues [61]. In 2010, China, the United States, Brazil, India, and Germany were the top five countries for antimicrobial consumption in food animals, accounting for 23%, 13%, 9%, 3%, and 3% of total consumption, respectively, with projections showing increases for China (30%), the United States (10%), Brazil (8%), India (4%), and Mexico (2%) by 2030 [61].

**Figure 10:**
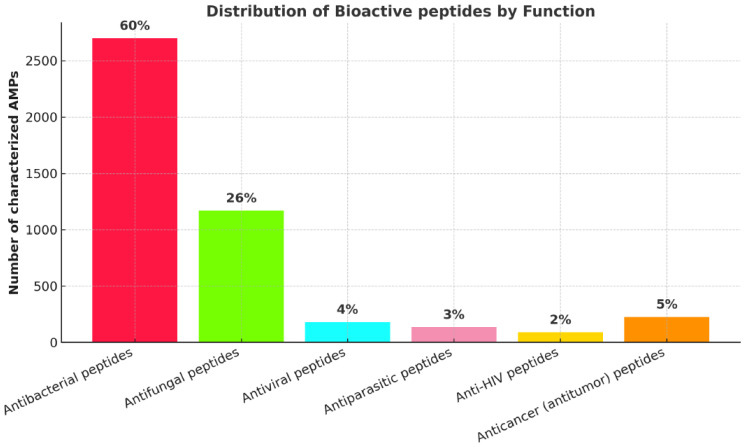
Statistics of the main functions of antimicrobial peptides.

Within this context, AMPs have emerged as noteworthy alternatives to conventional antimicrobial drugs. One of the most significant advantages of AMPs over traditional antibiotics lies in their mechanism of action. While conventional antibiotics typically target specific bacterial metabolic pathways or growth processes through interaction with protein receptors, AMPs primarily interact with the negatively charged bacterial cell wall and membrane due to their positive charge and hydrophobicity [108]. This fundamental difference makes it considerably more difficult for microbes to develop resistance to AMPs, as doing so would require complex modifications to membrane and cell wall components [108]. Additionally, unlike many primarily bacteriostatic antibiotics (inhibiting bacterial growth), AMPs often exhibit bactericidal properties, directly killing pathogens (Figure 10) [61].

AMPs typically exhibit broad-spectrum activity against both Gram-positive and Gram-negative bacteria, as well as fungi, viruses, and parasites [107]. Many AMPs are also effective against antibiotic-resistant bacteria due to their unique mechanisms of action, providing a solution for infections caused by multidrug-resistant pathogens [107]. Additionally, AMPs generally exhibit low toxicity toward eukaryotic cells, making them safer for use in food-producing animals compared to some antibiotics [107]. Regardless it is important to note that the haemolytic effects of 24 structurally related peptides were assessed in erythrocytes from four species: human, canine, bovine, and rat, as reported by Greco et al. Among the peptides tested, four exhibited strong haemolytic activity, causing nearly total lysis at 80 µM in human, rat, and dog erythrocytes. However, most peptides induced haemolysis of less than 40% at 150 µM. Canine erythrocytes demonstrated the highest sensitivity, with human and rat cells falling in the intermediate range. In contrast, bovine erythrocytes were relatively resistant to lysis, with only 12 of the compounds causing over 8% haemolytic activity at 150 µM. Several natural and synthetically improved AMPs also display ability to disrupt mammalian cells, thereby exerting an unwanted collateral toxicity [109].

Beyond their direct antimicrobial activity, AMPs demonstrate a remarkable range of additional biological functions that enhance their therapeutic potential. They can act as immunomodulators, influencing processes such as phagocytosis, cytokine release, and cell apoptosis [61]. They also function as chemotactic factors, facilitating the recruitment and aggregation of immune cells at sites of inflammation [61]. Furthermore, AMPs promote angiogenesis and wound healing by releasing cytokines and stimulating cell proliferation, thereby providing comprehensive support for recovery from infection [61].

While antibiotics have well-defined mechanisms of action and generally good bioavailability and stability, their use is increasingly challenged by the rise of microbial resistance. They often act slowly, may disrupt the host microbiota, and can cause inflammatory side effects. The development of new antibiotics is hindered by complex safety testing and challenges in the discovery process (Table 8). Furthermore, their effectiveness is threatened by widespread use and growing resistance, highlighting the urgent need for alternative strategies and more responsible application.

Farm animals harbor a diverse array of AMPs that contribute to their natural defense systems. In bovines, significant AMPs include indolicidin, bovine myeloid antimicrobial peptides (BMAPs), tracheal antimicrobial peptide, lingual antimicrobial peptide, and various bovine neutrophil β-defensins [61]. Equines produce unique AMPs such as equine cathelicidins (eCATH1, eCATH2, eCATH3), equine neutrophil antimicrobial peptides (eNAP-1, eNAP-2), and equine hepcidin [61]. Porcine species generate protegrins, PR-39, prophenin-1, cecropin P1, and porcine myeloid antimicrobial peptides, while ovine and caprine animals produce various cathelicidins and β-defensins [61].

Specific bovine AMPs have shown particular efficacy against bovine respiratory disease (BRD) pathogens. BMAP-28, a synthetic BMAP-28 analog called Syn-1, and bactenecin 5 (Bac-5) have demonstrated significant antibacterial activity against *Mannheimia haemolytica*, a primary bacterial pathogen in BRD. BMAP-28 has also shown effectiveness against bovine herpes virus-1 and bovine respiratory syncytial virus, indicating potential broad-spectrum activity against both bacterial and viral BRD pathogens [45]. Furthermore, when BMAP-28 and Bac-5 were used in combination, a synergistic effect was observed, allowing for significantly reduced concentrations of both peptides while maintaining antimicrobial efficacy. This combination approach could potentially address concerns about cytotoxicity that occur at higher concentrations of individual AMPs [45].

A particularly prospective antimicrobial peptide for livestock applications is thanatin, which has shown substantial antibacterial efficacy against livestock pathogens. Research by Javadmanesh et al. (2021) [115] demonstrated that thanatin exhibited strong antimicrobial activity against various livestock pathogens with minimum inhibitory concentration (MIC) values ranging from 6.25 to 100 μg/mL. The peptide showed especially potent activity against *Escherichia coli* O157:H7 and *Salmonella* Enteritidis from cattle mastitis (MIC of 6.25 μg/mL). Additionally, thanatin displayed remarkable thermal stability at normal body temperatures of dairy cattle (37 °C) and avian species (42 °C), maintaining its structural integrity and antimicrobial function, which is a crucial characteristic for applications in animal health [115].

Recent research has also shown that antimicrobial peptides can influence the rumen microbiome and metabolome, thereby impacting cattle performance. In a study involving castrated bulls, supplementation with antimicrobial peptides improved daily weight gain, carcass weight, and net meat weight in the experimental animals [116]. The study also found that antimicrobial peptides increased the diameter of the rumen papillae and micropapillary density, which could enhance nutrient absorption [116]. Furthermore, the content of digestive enzymes such as protease, xylanase, and β-glucosidase was higher in the antimicrobial peptide-supplemented group, potentially improving feed utilization [116]. Metagenomic analysis revealed that antimicrobial peptides had a specific antimicrobial mechanism, effectively killing viruses and harmful bacteria while sparing beneficial bacteria [116]. Importantly, the study demonstrated that antimicrobial peptides could enhance immunity without developing drug resistance [116].

Studies in pigs have also provided encouraging results for the application of AMPs in livestock production. A study examining porcine intestinal antimicrobial peptide (PIAP) as an in-feed antibiotic alternative showed beneficial effects on intestinal morphology, digestive enzymes, immunity, and gut permeability in weaned piglets. Specifically, a relatively low dose of PIAP (400 mg/kg from day 1 to 24; 300 mg/kg from day 25 to 37) significantly improved intestinal villus height-to-crypt depth ratio, increased the activity of digestive enzymes including maltase, lactase, and sucrase, and enhanced secretory immunoglobulin A (SIgA) levels compared to control groups [117]. Additionally, PIAP supplementation was associated with increases in beneficial gut bacteria like *Lactobacillus reuteri*, which showed positive correlations with improved digestive enzyme activities and SIgA levels [117].

Comprehensive meta-analyses of antimicrobial peptides in piglets have provided further evidence of their efficacy as alternatives to antibiotics [118,119]. The analysis included data from 56 trials involving 4067 piglets and revealed that AMPs significantly improved healthy piglets’ average daily gain (ADG), average daily feed intake (ADFI), gain: feed ratio (G/F), immunoglobulin levels (IgM and IgG), and intestinal villus height: crypt depth ratio (V/C) compared to negative control groups [118]. In infected piglets, AMPs significantly increased ADG, ADFI, G/F, and V/C of the jejunum and ileum, while notably decreasing diarrhea rates [118]. When compared to antibiotics, AMPs showed slightly weaker effects in healthy piglets but demonstrated similar efficacy to antibiotics in infected piglets [118]. The optimal dose of highly purified AMPs was determined to be approximately 0.01% [118].

In livestock production, insect meals have emerged as another potential source of AMPs, providing both a protein supplement and potential antimicrobial benefits. Studies have shown that insect meals can positively influence gastrointestinal microbiota, strengthen immunity, and increase antibacterial activity in farm animals. Animals fed insect meals have demonstrated improved intestinal health, characterized by changes in gut microflora, an enhanced immune response, and greater resistance to pathogens [110]. Insects produce the largest number of identified antimicrobial peptides in the animal kingdom, making them an excellent source for novel antimicrobial compounds. These insect-derived AMPs are categorized into major groups, including defensins (cysteine-rich peptides), cecropins (α-helical AMPs), moricins, proline-rich peptides, and glycine-rich peptides, each with distinctive structures and mechanisms of action [110].

The black soldier fly (*Hermetia illucens*) has garnered particular attention as a sustainable source of AMPs. These insects naturally thrive in environments rich in microbes and have evolved robust antimicrobial defense systems. Xia et al. (2021) [120] describe how antimicrobial peptides from black soldier fly have great potential as alternatives to antibiotics for prophylaxis and treatment of diseases in animals due to their extensive antimicrobial properties and lower tendency to induce resistance. Their study highlights that black soldier fly larvae can participate in a circular economy by digesting organic waste and then promoting the growth performance of domestic animals fed the larvae [120]. This approach establishes a sustainable cycle in which waste is converted into valuable antimicrobial protein sources for livestock.

### 4.2. Antifungal and Antiparasitic Properties of AMPs and Their Relevance

A significant advantage of many AMPs is their broad-spectrum activity not only against bacteria but also against fungi, which represents an important consideration for livestock health management. Buda De Cesare et al. [50] emphasize that while current research often focuses on antibacterial peptides, there are many with antifungal properties that represent important potential additions to the antimicrobial arsenal.

Fungal infections in livestock can cause significant economic losses and health problems, yet they have received less attention compared to bacterial infections. Cathelicidin-inspired “PepBiotics” have shown remarkable efficacy against fungi, including azole-resistant strains, suggesting their potential in addressing these challenges [121].

Understanding the antifungal mechanisms of cathelicidin-inspired AMPs is crucial for their application in livestock disease management. Research indicates that AMPs kill target cells through diverse mechanisms, primarily by disrupting the membrane and cell wall. Still, they can also target key cellular processes, including DNA and protein synthesis, protein folding, cell wall synthesis, and enzymatic activity [121]. The broad-spectrum antifungal activity of cathelicidins has been demonstrated against medically relevant fungal species, including species of *Aspergillus*, *Candida*, *Cryptococcus*, *Fusarium*, *Malassezia*, and *Talaromyces* [121].

A study by van Eijk and colleagues demonstrated that cathelicidin-inspired antimicrobial peptides termed “PepBiotics” showed potent inhibitory activity against a variety of filamentous fungi and yeasts at low concentrations (≤1 μM), including azole-resistant *Aspergillus fumigatus* isolates [121]. Cytotoxicity testing of PepBiotics on primary human nasal epithelial cells has shown that many of these compounds have no significant cytotoxic effect at concentrations effective against pathogens [121]. This favorable safety profile further supports their potential for livestock applications, where safety for the treated animals and the absence of harmful residues in animal products are crucial considerations.

Regarding antiparasitic activity, AMPs have also demonstrated significant potential as alternatives to conventional antiparasitic drugs, which is particularly relevant in livestock production, where parasitic diseases can cause substantial economic losses. Parasitic protozoa can cause diseases in humans and animals through various routes, including direct contact, contaminated water, soil, and food [47]. With the increasing prevalence of parasite drug resistance, the need for new treatments has intensified. Antiparasitic peptides exhibit killing effects against parasites that cause diseases such as malaria and leishmaniasis [47], and AMPs, including cathelicidin and temporins-SHd, demonstrate high inhibitory activity against various parasites [47].

The peptide Jellein, derived from bee royal jelly and a 4-amino acid AMP called KDEL (lysine, aspartic acid, glutamic acid, and leucine), have shown significant effects against *Leishmania* parasites [60]. However, it is worth noting that their mechanisms of action differ. Cyanobacterial peptides differ from higher-eukaryote AMPs because their antiparasitic action depends on specific protein targets. This allows these peptides to distinguish accurately between target parasites even when they belong to the same family or genus [60]. The distinctive mechanism of action of cyanobacterial peptides provides a precision tool for addressing specific parasitic infections in livestock, potentially enabling more targeted treatment approaches compared to conventional broad-spectrum antiparasitic drugs. This specificity could be particularly valuable in minimizing disruption to beneficial microbiota in the host animal while effectively eliminating pathogenic parasites.

### 4.3. Addressing Challenges: Enhancing AMP Stability via Self-Assembly

Despite their considerable potential, several challenges have limited the clinical translation of AMPs. Stability is a primary concern, as AMPs are often rapidly cleared from the bloodstream through enzymatic degradation, renal filtration, and uptake by the reticuloendothelial system [108]. To address this, researchers have explored nanotechnology approaches, particularly self-assembly strategies, to enhance the stability of AMPs and prolong their half-lives [108]. For example, self-assembled peptide CPC-1 demonstrated a fourfold increase in half-life retention at infection sites compared to non-self-assembled variants [108]. Similarly, peptide PTP-7 showed significant improvements in serum stability, extending from 2 h to more than 5 h through self-assembly technology [108].

Recent innovations in self-assembly technology have led to the development of AMPs that can transform from nanoparticles to nanofibers in response to bacterial cell wall components like lipopolysaccharides (LPS) and lipoteichoic acid (LTA) [108]. These transformations enhance stability and enable bacterial capture through nanofibrous networks, complementing traditional membrane-disruption mechanisms [108]. For instance, the self-assembled peptide FFN can form nanoparticles at concentrations as low as 35.46 μM and dynamically transform into nanofibers when exposed to bacteria, resulting in significant improvements in stability against trypsin and tissues compared to non-assembled peptides [108].

Several AMP-based products have already reached clinical application, including dalbavancin, daptomycin, and nisin, demonstrating the feasibility of translating these compounds into commercial therapeutics [108]. In veterinary medicine, AMPs show particular prospects for treating mastitis, a common and economically significant infection in dairy cattle [108]. Studies using mouse models have shown that self-assembled AMPs can effectively alleviate mastitis caused by MDR *Escherichia coli* and *Staphylococcus aureus* by eliminating pathogenic bacteria, reducing inflammatory markers, and preserving mammary tissue integrity [108].

One significant advantage of AMPs is that their non-specific mechanism of action, through electrostatic interactions with the microbial membrane and cell wall, reduces the opportunities for resistance development, unlike conventional antibiotics [110]. Practical applications in farm settings have already shown that supplementing animal feed with AMPs can reduce the incidence of diarrhea, improve growth performance, and enhance intestinal function in weaned piglets, demonstrating their potential as viable alternatives to conventional antibiotics in commercial production settings [117].

Continued advancements in peptide design, self-assembly technologies, delivery systems, and combination approaches will likely enhance the stability, efficacy, and cost-effectiveness of AMP-based therapeutics. By providing effective antimicrobial, antifungal, and antiparasitic solutions with reduced risk of resistance development, AMPs represent a valuable tool in preserving the efficacy of conventional drugs for critical applications while maintaining animal health and productivity in sustainable agriculture systems.

## 5. Nano-Enabled Polymeric Systems for Pathogen Control in Animal Farming

The application of nanotechnology in veterinary medicine has been expanding as researchers explore various nano-systems, including liposomes, metallic nanoparticles, polymeric micelles, nanospheres, functionalized fullerenes, carbon nanotubes, dendrimers, polymer-coated nanocrystals, and nanoshells [122]. These nanotechnology applications are particularly valuable for animal health and production, as they can help solve problems related to delivering antibiotics that suffer from poor bioavailability and numerous side effects. Nanoparticles are characterized by their small size and large surface area-to-mass ratio, making them effective delivery systems for antimicrobials in the treatment of microbial diseases by enhancing therapeutic effects while minimizing side effects [122].

The veterinary applications of various nanoparticles extend to different animal farming contexts, with research addressing therapeutic, preventive, and diagnostic indications [122]. Nanoparticles used for therapeutic purposes in veterinary medicine can control drug release, target neurotic sores effectively, improve medication bioavailability, and reduce required treatment dosages, which has significant economic benefits. They also enable the treatment of intracellular pathogens like *Brucella* and *Leishmania*, as well as multi-antibiotic-resistant pathogens such as methicillin-resistant *Staphylococcus aureus* (MRSA) [122].

Nanoparticles in association with antimicrobial peptides, termed NanoAMPs, offer versatile structures for the protection and controlled release of AMPs [123]. These NanoAMPs can overcome challenges associated with traditional AMP applications, such as reduced defensive activity due to degradation when exposed to microbial proteases or environmental conditions [123]. In the context of animal farming, NanoAMPs could provide sustained antimicrobial action against common pathogens while minimizing the development of resistance.

Polymers, including polyethylene glycol (PEG), poly lactic-co-glycolic acid (PLGA), chitosan, poly-L lysine (PLL), and hyper-branched polyglycerol (HPG), have been extensively employed in AMP drug delivery systems. These polymers act as carriers, protecting peptides from degradation and facilitating their delivery across the cell wall and cellular membrane to specific target sites [112]. Polymer-AMP conjugates can significantly improve antimicrobial efficacy while reducing potential toxicity concerns.

The use of polymer nanocarriers for drug delivery offers numerous advantages, including protection from degradation, controlled release, and increased bioavailability in tissues and cells [4]. For instance, chitosan nanoparticles (CS NPs) have been widely studied for delivering various essential oils with antimicrobial properties. These CS-NPs systems demonstrate improved efficacy against pathogenic bacteria while preserving beneficial intestinal flora bacteria such as *Lactobacillus* spp. [4]. Studies conducted on poultry show that nanoencapsulation of essential oils in chitosan carriers improves body weight gain, feed conversion ratio, and feed intake in broiler chickens [4].

Chitosan (CS) is a natural, positively charged cationic polymer with a broad spectrum of antimicrobial properties that works by associating with negatively charged particles on bacterial cell surfaces. However, its antibacterial ability on its own is limited [124]. When combined with gold nanoparticles (AuNPs) through electrostatic interactions, CS-AuNPs show enhanced antimicrobial activity by disrupting the bacterial cell wall and membrane [124]. This synergistic effect between the gold core and surface ligands creates a more potent antimicrobial system that effectively kills bacteria, including methicillin-resistant *Staphylococcus aureus* (MRSA), which is particularly problematic in animal agriculture [124].

Several polymeric nanoparticles functionalized with AMPs have demonstrated remarkable potential for pathogen control. For instance, PLGA nanoparticles loaded with LL37 (a human cathelicidin AMP) have shown significant antimicrobial activity against *Escherichia coli* while accelerating wound healing [112]. PLGA has been recognized as a biocompatible, biodegradable polymer approved by regulatory agencies for biomedical applications, making it suitable for veterinary use [4]. The release of essential oils from PLGA nanoparticles typically follows a biphasic pattern, characterized by an initial burst effect, followed by a slower, sustained release [4].

A novel nano-antimicrobial polymer engineered with chitosan nanoparticles and bioactive peptides has demonstrated significant potential as a food biopreservative for controlling foodborne pathogens. In a recent study, researchers developed chitosan nanoparticles with the antimicrobial peptide microcin J25 (CNMs), which showed excellent bactericidal activity against *E. coli* O157, a critical foodborne pathogen that causes food contamination, diarrhea, and death in humans, neonates, and weaned animals [125]. The CNMs protected against *E. coli* O157-induced intestinal barrier dysfunction by increasing transepithelial electrical resistance, decreasing lactate dehydrogenase release, and promoting the expression of tight junction proteins, such as occludin. Additionally, they ameliorated inflammation by modulating inflammatory cytokines and inhibiting the activation of mitogen-activated protein kinase and NF-κB [125].

Metal and metal oxide nanoparticles, particularly silver nanoparticles, have exhibited strong antimicrobial properties by disrupting bacterial cell walls and membranes. When functionalized with AMPs, these nanoparticles have demonstrated potent effects in killing mycobacteria without causing cytotoxicity or DNA damage [112]. Silver nanoparticles (AgNPs) are known to damage the structure of the bacterial cell wall and membrane and depress the activity of membranous enzymes. Several studies have shown that AgNPs can interact with sulfur-containing proteins in bacterial membrane and with phosphorus groups in cell DNA, preferably attacking the respiratory chain and cell division processes [11]. Gold nanoparticles (AuNPs), while not inherently antimicrobial, can be modified with ligands to achieve potent bactericidal effects through direct multivalent interactions with the bacterial membrane [124]. The morphology of bacterial strains treated with CS-AuNPs shows significant disruption, characterized by cell membrane collapse and rupture [124].

Polymeric nanofibers incorporating AMPs have also shown the capability for pathogen control. These nanofibers can be designed to release AMPs over extended periods, maintaining their antimicrobial activity. For example, plantaricin 423 and ST4SA (bacteriocins with antimicrobial properties) incorporated into poly D, L-lactide and polyethylene oxide nanofibers have demonstrated effective control of microbial infections [112]. Similarly, cellulose nanofibers incorporating essential oils, such as thyme, have demonstrated antibacterial properties against both Gram-positive and Gram-negative bacteria [4].

Liposomal formulations of AMPs represent another prospective approach for pathogen control in animal farming. Liposomes can extend the drug half-life, improve biocompatibility, and reduce the toxicity of encapsulated AMPs [112]. For instance, liposome-encapsulated nisin (an antimicrobial peptide commonly used in food preservation) has shown enhanced efficacy against *Listeria monocytogenes* compared to free nisin [112]. Nanostructured lipid carriers loaded with essential oils can penetrate bacterial cells, disrupt biomembranes, release polypeptides into the medium, and decrease the cellular ATP content [4]. Lipid-based nanocarrier systems have been shown to provide sustained release of essential oils for improved therapeutic efficacy [4].

Hydrogels incorporating AMPs have demonstrated potential for wound healing applications that could benefit animal agriculture. These hydrogels can maintain antimicrobial activity while providing a moist environment conducive to healing [112]. For example, gelatin methacryloyl hydrogels loaded with antimicrobial peptides have shown biocompatibility, biodegradation, and promotion of wound healing in vivo [112]. Similarly, sodium alginate films loaded with essential oils have shown potential for wound dressings with antimicrobial properties against both Gram-positive and Gram-negative bacteria [4]. Hydrogel dressings can effectively soak up inflammatory exudates from wounds, preventing their accumulation and accelerating healing [124], which is particularly important in animal farming, where wound management is challenging.

In the field of nano-vaccines and nano-adjuvants, nanoparticles are increasingly used in veterinary vaccine production due to their ability to improve immunological responses, serving as adjuvants that allow for slow release of antigens, which enhances vaccine performance [122]. These include nano-emulsion vaccines, PLGA nanoparticle-loaded vaccines, chitosan nanoparticle vaccines, and gold nanoparticle-based vaccines for various animal diseases [122].

The application of NanoAMPs in agriculture could significantly impact food security by controlling phytopathogens and pests that cause substantial yield losses in major crops [123]. The same principles could be applied to animal farming, where infectious diseases can lead to significant economic losses and compromise animal welfare.

Recent advances in antimicrobial polymer-based assemblies have further expanded the toolkit available for pathogen control in animal farming. Carmona-Ribeiro and Araújo (2021) [111] highlight that cationic antimicrobial polymers (APs) offer several advantages over AMPs, including lower manufacturing costs, resistance to proteolytic degradation, and the ability to be produced on a large scale following industrial synthetic protocols. These polymers typically contain cationic moieties such as quaternary ammonium, sulfonium, guanidinium, or phosphonium groups, along with hydrophobic components that facilitate interaction with the bacterial cell wall and membrane [111].

Zein, a protein derived from maize, has also been explored as a nanocarrier for essential oils in animal farming applications. Zein nanoparticles loaded with essential oils have shown bactericidal activity against pathogenic bacteria in fish, offering a potential alternative to antibiotics such as oxytetracycline, florfenicol, amoxicillin, and erythromycin in aquaculture [4]. These formulations demonstrate high encapsulation efficiency and maintain stability during extended storage periods [4].

As a result, nano-enabled polymeric systems constitute a novel and prospective platform for controlling pathogens in animal farming by enabling targeted delivery, boosting antimicrobial efficacy, and minimizing ecological impact. The functionalization of such systems with AMPs via incorporation into various biomaterials, such as nanoparticles, liposomes, polymers, and hydrogels, further augments their therapeutic potential and stability [40]. Nonetheless, realizing their full potential requires overcoming several technical and regulatory challenges, including upscaling, economic feasibility, and environmental safety. Furthermore, their clinical applicability remains to be validated through comprehensive in vivo studies [123,126]. Finally, there remains a lack of essential data regarding resistance development rates and species-specific dosing strategies [4].

## 6. Advantages and Future Perspectives

One of the key advantages of AMPs is their potent antimicrobial activity against a broad spectrum of pathogens relevant to farm animal health. Studies have demonstrated their effectiveness against various Gram-positive bacteria, including *Staphylococcus aureus*, *Bacillus cereus*, *Bacillus subtilis*, *Listeria monocytogenes*, and *Enterococcus faecalis* [4]. They also show efficacy against Gram-negative bacteria such as *Escherichia coli*, *Salmonella* serovars (including *Salmonella *Typhimurium and *Salmonella* Enteritidis), *Proteus mirabilis*, *Yersinia* spp., *Klebsiella pneumoniae*, *Shigella boydii*, *Acinetobacter anitratus*, and *Pseudomonas aeruginosa* [4].

The mechanisms of action of AMPs are diverse and often involve targeting the bacterial membrane and cell wall [26,43], which contributes to their effectiveness against multidrug-resistant pathogens. Furthermore, AMPs possess immunomodulatory properties that extend beyond direct killing [15], enhancing their potential as therapeutic agents. For instance, porcine β-defensins (pBDs) have demonstrated both direct antimicrobial effects and the ability to modulate immune responses [15]. Recent advances in research have revealed that certain AMPs can overcome resistance mechanisms that affect conventional antibiotics, including vancomycin resistance in *Staphylococcus aureus* [43], making them valuable candidates for addressing the growing AMR crisis.

Despite these significant advantages, several challenges must be addressed before AMPs can be widely adopted in livestock production systems. While favorable in vitro results have been obtained for specific peptides, such as pBDs [15], more comprehensive quantitative studies and in vivo evaluations are needed before commercial application [4].

The stability and delivery of AMPs present another significant hurdle. These peptides can be susceptible to degradation by proteases in the gastrointestinal tract and may have limited bioavailability when administered orally [43]. Mishra et al. [17] note that AMPs face several limitations that hinder their clinical applications, including instability in clinically relevant environments, susceptibility to protease degradation, toxicity to host cells, and high development and production costs. To address these limitations, innovative delivery systems are being explored. Nanodelivery technologies, which have proven effective for other antimicrobial agents like essential oils, could potentially enhance the dispersibility, stability, and release kinetics of AMPs [4]. Such approaches could improve the efficacy and practical application of AMPs in livestock production systems. Pei et al. designed and synthesized a new cyclic AMP called C-LR18. This compound retains the low hemolysis and low cytotoxicity benefits of the original peptide. C-LR18 exhibits enhanced antibacterial activity and stability compared to its predecessor, with a longer half-life observed in rats, both in vivo and in vitro. Additionally, it demonstrated therapeutic effects against *E. coli* infections in mice, positioning it as a promising candidate for development into a therapeutic drug for bacterial diseases in both human and veterinary applications [127]. Cyclic AMPs exhibit greater resistance to enzymatic digestion, potentially increasing their bioavailability and prolonging their half-life in vivo. Additionally, they are less prone to conformational changes that may diminish their antimicrobial effectiveness [128].

The cost-effectiveness of AMP production also remains a concern for commercial applications [43]. Current production methods may be expensive compared to conventional antibiotics, potentially limiting widespread adoption. However, advances in biotechnology and the increasing scale of production could potentially reduce costs over time. Additionally, economic assessments of AMPs should consider not only direct production costs but also the broader economic benefits of reduced antimicrobial resistance [13].

The application of advanced techniques, including computational approaches and genomic tools, could accelerate the discovery and optimization of new AMPs with enhanced properties [43]. Additionally, interdisciplinary approaches combining insights from microbiology, immunology, and animal science could advance our understanding of AMP applications in livestock production systems. In the last twenty years, advanced computational techniques, such as database management, artificial intelligence (AI), molecular docking, and molecular dynamics (MD) simulations, have significantly influenced the progress of AMP development. Initial endeavors, like quantitative structure–activity relationship (QSAR) models, established a basis for AI-assisted AMP design by examining structure–activity correlations. The ongoing enhancement of AMP database models using machine learning (ML) algorithms has enabled strong predictions of AMP bioactivity by revealing hidden patterns in complex datasets. Additionally, deep learning (DL)-based generative models have enabled the de novo creation of peptides, paving the way for the discovery of new AMPs. Meanwhile, MD simulations and molecular docking have deepened our understanding of the mechanisms of action of AMPs, providing essential atomic-level insights into how peptides interact with targets such as bacterial membranes and proteins. This knowledge lays a solid theoretical groundwork for the design and optimization of AMPs. Overall, the integration of computational methods in AMP design has undergone rapid evolution over the past two decades, propelling AMP development toward improved efficiency and precision [129].

AMPs with broad-spectrum antibacterial, antiviral, antifungal, and anticancer properties are expected to serve as alternative antibiotics through AMP-based therapies. Currently, the FDA has approved several AMPs for antibacterial treatment, while others are in clinical development. Despite their potential, AMPs face challenges. Intravenous administration is limited by their short half-life and enzymatic degradation in blood plasma. Oral administration is hindered by pre-systemic peptide degradation and inadequate intestinal mucosa penetration. Clinical trials primarily restrict the use of AMPs to topical methods due to enzymatic degradation, systemic toxicity, and rapid clearance by the liver and kidneys. Therefore, localized application via topical creams is the most common route for AMPs. To enhance AMP delivery, polymeric materials such as hydrogels, chitosan, and hyaluronic acid are utilized. AMPs can be either covalently bonded or non-covalently encapsulated in these systems. PEGylation reduces non-specific tissue uptake and cellular toxicity while enhancing blood half-life and stability against proteolytic degradation. Additionally, conjugating AMPs to hyperbranched polyglycerol (HPG) increases their antimicrobial effectiveness. Lipids and surfactants can protect peptides from harsh conditions. Other explored materials include mesoporous silica particles, quantum dots, gold and silver nanoparticles, titanium, graphene, and carbon nanotubes. Finally, the high cost of peptide production challenges the advancement of AMPs, but recombinant production in prokaryotic or yeast systems through genetic engineering can alleviate this issue [130].

## 7. Conclusions

Antimicrobial peptides represent a prospective alternative to conventional antibiotics in livestock production, with potential benefits for animal health, food safety, and addressing antimicrobial resistance. Their broad-spectrum antimicrobial activity, immunomodulatory properties, and ability to overcome resistance mechanisms make them attractive candidates for future applications. However, achieving their full potential will require addressing challenges related to delivery, stability, production costs, and in vivo efficacy.

Strategies such as lipidation, modifying peptides into peptidomimetics, and combining AMPs with other peptides or conventional antibiotics offer opportunities to enhance their stability, safety, and efficacy, making them more suitable for clinical applications in livestock. The authors emphasize that the quest for new alternatives to treat infections should focus not only on AMPs but also on their combination with other active molecules.

Ultimately, evaluating both molecular outcomes and behavioral outcomes is necessary to fully assess the safety and efficacy of AMPs as alternatives to traditional antimicrobials in livestock sectors. With continued research and development efforts, AMPs could play a significant role in sustainable livestock production systems while contributing to global efforts to combat antimicrobial resistance.

## Figures and Tables

**Table 1:** Examples of AMP Sequences from Livestock Species with Corresponding UniProt Entries [18].

AMP	Source Organism	Uniprot Entry Name	Sequence
PR-39	*Sus scrofa* (Pig)	PR39_PIG	METQRASLCLGRWSLWLLLLGLVVPSASAQALSYREAVLRAVDRLNEQSSEANLYRLLELDQPPKADEDPGTPKPVSFTVKETVCPRPTRQPPELCDFKENGRVKQCVGTVTLNPSIHSLDISCNEIQSVRRRPRPPYLPRPRPPPFFPPRLPPRIPPGFPPRFPPRFPGKR
Beta-defensin 1	*Sus scrofa* (Pig)	DEFB1_PIG	MRLHRLLLVFLLMVLLPVPGLLKNIGNSVSCLRNKGVCMPGKCAPKMKQIGTCGMPQVKCCKRK
Beta-defensin 10	*Bos taurus* (Bovine)	DFB10_BOVIN	MRLHHLLLLLLLVVLSSGSGFTQGVRSYLSCWGNRGICLLNRCPGRMRQIGTCLAPRVKCCR
Cathelicidin-1	*Gallus gallus* (Chicken)	CTHL1_CHICK	MLSCWVLLLALLGGACALPAPLGYSQALAQAVDSYNQRPEVQNAFRLLSADPEPGPNVQLSSLHNLNFTIMETRCQARSGAQLDSCEFKEDGLVKDCAAPVVLQGGRAVLDVTCVDSMADPVRVKRVWPLVIRTVIAGYNLYRAIKKK
Cathelicidin-2	*Gallus gallus* (Chicken)	CTHL2_CHICK	MLSCWVLLLALLGGVCALPAPLSYPQALIQAVDSYNQRPEVQNAFRLLSADPEPGPGVDLSTLRALNFTIMETECTPSARLPVDDCDFKENGVIRDCSGPVSVLQDTPEINLRCRDASSDPVLVQRGRFGRFLRKIRRFRPKVTITIQGSARFG
Cathelicidin-4	*Bos taurus* (Bovine)	CTHL4_BOVIN	MQTQRASLSLGRWSLWLLLLGLVVPSASAQALSYREAVLRAVDQLNELSSEANLYRLLELDPPPKDNEDLGTRKPVSFTVKETVCPRTIQQPAEQCDFKEKGRVKQCVGTVTLDPSNDQFDLNCNELQSVILPWKWPWWPWRRG
Cathelicidin-6	*Bos taurus* (Bovine)	CTHL6_BOVIN	METQRASLSLGRWSLWLLLLGLALPSASAQALSYREAVLRAVDQFNERSSEANLYRLLELDPPPKEDDENPNIPKPVSFRVKETVCPRTSQQPAEQCDFKENGLVKQCVGTVTLDAVKGKINVTCEELQSVGRFKRFRKKFKKLFKKLSPVIPLLHLG
Gallinacin-1	*Gallus gallus* (Chicken)	GLL1_CHICK	MRIVYLLLPFILLLAQGAAGSSQALGRKSDCFRKSGFCAFLKCPSLTLISGKCSRFYLCCKRIWG
Hepcidin	*Larimichthys crocea* (Large yellow croaker)	HEPC_LARCR	MKTFSVAVAVAVVLAFICLQESSAVPANEEQELEQQIYFADPEMPVESCKMPYYMRENRQGSPARCRFCCRCCPRMRGCGICCRF
LEAP 2	*Sus scrofa* (Pig)	LEAP2_PIG	MWHLKLFAVLVICLLLAVQVHGSPIPELSSAKRRPRRMTPFWRAVSLRPIGASCRDDSECLTRLCRKRRCSLSVAQE
Oncorhyncin-1	*Salmo gairdneri* (Rainbow trout)	ONC1_ONCMY	SKGKKANKDVELARG
Ostricacin-1	*Struthio camelus* (Common ostrich)	OSTR1_STRCA	LFCRKGTCHFGGCPAHLVKVGSCFGFRACCKWPWDV
Piscidin-3	Hybrid *Morone chrysops* x *Morone saxatilis* (Hybrid White bass x Striped bass)	PISC3_MORCS	FIHHIFRGIVHAGRSIGRFLTG
Protegrin-1	*Sus scrofa* (Pig)	PG1_PIG	METQRASLCLGRWSLWLLLLALVVPSASAQALSYREAVLRAVDRLNEQSSEANLYRLLELDQPPKADEDPGTPKPVSFTVKETVCPRPTRQPPELCDFKENGRVKQCVGTVTLDQIKDPLDITCNEVQGVRGGRLCYCRRRFCVCVGRG

**Table 2:** Examples of Antimicrobial Peptides for Use in Aquaculture: Sources and Target Pathogens.

Peptide	Source Organism	Target Pathogen	Reference
Algal AMPs (various)	Marine photosynthetic organisms	Gram-positive & Gram-negative bacteria, parasites	[19]
Hepcidin	Fish	Bacteria, fungi, parasites	[16]
Lc149	Large Yellow Croaker (*Larimichthys crocea*)	*Escherichia coli*, *Vibrio harveyi*, fish parasites	[20]
Lc1687	C-terminal fragment of a Ferritin H in *Larimichthys crocea*	Gram-positive & Gram-negative bacteria	[21]
LEAP-2	Golden pompano (fish)	*Edwardsiella tarda* and *Streptococcus agalactiae*	[22]
NK-lysin	Atlantic salmon (*Salmo salar*)	*Piscirickettsia salmonis*, *Flavobacterium psychrophilum*	[23]
Oncorhyncin III	Non-histone chromosomal protein H6 from Rainbow Trout	Gram-positive & Gram-negative bacteria	[24]
Piscidin	Fish	Bacteria, viruses, fungi, parasites	[16]

**Table 3:** Examples of Antimicrobial Peptides for Use in Poultry: Sources, Target Pathogens, and Intended Applications.

Peptide	Source Organism	Target Pathogen	Target	Reference
A11	Modified Acidocin J1132β (from *L. acidophilus*)	*Salmonella* Typhimurium	Poultry (Food chain)	[26]
ABD1	Chicken	Gram-positive & Gram-negative bacteria	Chicken	[27,28]
Brevilaterins	*Brevibacillus laterosporus*	Bacteria, Fungi	Livestock (general, incl. aquaculture, poultry, and swine)	[29]
C2-2	Modified chicken CATH-2	Multidrug-resistant *E. coli*	Chicken	[30]
CATH-1(6–26)	Chicken	Gram-positive & Gram-negative bacteria	Chicken	[26]
Enterocin A and B	*Enterococcus faecium* from poultry	*Clostridium perfringens*	Poultry	[25]
Fowlicidins (cathelicidins from chicken)	Chicken	Gram-positive & Gram-negative bacteria	Chicken	[31]
OaBac5mini	*E. coli* recombinant system	*Salmonella* Pullorum	Chicken	[32]
Ostricacins	Ostrich	Bacteria	Ostrich	[28]
P1 (NPSRQERR)	*Lactobacillus rhamnosus* GG	Avian Pathogenic *E. coli* (APEC)	Chicken	[33]
Rabbit sacculus rotundus-derived AMP	Rabbit sacculus rotundus	vvIBDV (very virulent infectious bursal disease virus)	Chicken	[34]
Sophorolipids (SL1–SL4)	*Candida bombicola* and other yeasts	*Eimeria maxima* and *Clostridium perfringens*	Chicken	[35]

**Table 4:** Examples of Antimicrobial Peptides for Use in Swine Production: Sources, Target Pathogens, and Intended Applications.

Peptide	Source Organism	Target Pathogen	Target	Reference
Brevilaterins	*Brevibacillus laterosporus*	Bacteria, Fungi	Livestock (general, incl. aquaculture, poultry, and swine)	[29]
Cecropin AD	Synthetic hybrid (insect cecropins A & D)	*E. coli* (enterotoxigenic strain)	Pig (weaned piglets)	[36,37]
Epinecidin-1	*Epinephelus* *coioides*	MRSA	Pig	[38,39]
LEAP-2	Golden pompano (fish); Pig liver	*Salmonella* Typhimurium	Aquaculture species; Pig	[15]
PR-39	Pig (intestinal cathelicidin)	*Salmonella* Typhimurium	Pig (swine)	[40]
Porcine β-Defensins (pBDs)	Pig	*Escherichia coli* (ETEC—post-weaning diarrhea)	Pig (swine)	[41]
Protegrin-1 (PG-1)	Porcine leukocytes	Gram-positive & Gram-negative bacteria	Piglets	[28,42]

**Table 5:** Examples of Antimicrobial Peptides for Use in Ruminants: Sources, Target Pathogens, and Intended Applications.

Peptide	Source Organism	Target Pathogen	Target	Reference
BMAP-27	Bovine myeloid antimicrobial peptide	*E. coli*	Calves (cattle)	[42]
Bac-7	*Bos taurus*	*E. coli*, *Salmonella* Typhimurium	Cattle	[43]
Bacteriocins	Gram-positive & Gram-negative bacteria	Gram-positive & Gram-negative bacteria	Eggs, poultry, and dairy products	[26,44]
Cathelicidin 4	*Bos taurus*	Gram-positive & Gram-negative bacteria	Cattle	[28,45]
Cecropin B	Insect (moth peptide, via transgenic expression)	*Staphylococcus aureus* (mastitis pathogen)	Goat (dairy goats)	[46]
Indolicidin	Bovine neutrophils; *Bos taurus*	Gram-positive & Gram-negative bacteria	Calves (cattle)	[28,42]
Lfcin B (Lactoferricin B)	Cattle milk (lactoferrin fragment)	Broad-spectrum bacteria	Cattle, dairy products	[47]

**Table 6:** Examples of Antifungal Peptides for Use in Livestock: Sources, Target Pathogens, and Intended Applications.

Peptide	Source Organism	Target Pathogen	Target Animal	Reference
Fmoc-dipeptide 7a	Synthetic (modeled for veterinary pathogens)	*Aspergillus flavus*, *Aspergillus versicolor*, *Aspergillus candidus*	Cattle	[49]
Defensins	Mammalian immune cells, Plants	Broad-spectrum (bacteria, fungi, viruses)	Multiple livestock species	[48,50]
Piscidin	Fish	Bacteria, viruses, fungi, parasites	Aquaculture species	[16,48]
Hepcidin	Fish	Bacteria, fungi, parasites	Aquaculture species	[16]
SMAP-29	Sheep (*Ovis aries*)	*C. albicans*, *C. neoformans*, and *R. rubra*	Sheep	[48]
Cathelicidins	Sheep, Cattle, Pigs	Broad-spectrum: bacteria, fungi	Ruminants	[28,50]

**Table 7:** Examples of AMPs against parasites in Livestock: Sources, Target Pathogens, and Intended Applications.

Peptide	Source Organism	Target Pathogen	Target	Reference
Fabclavine	*Xenorhabdus szentirmaii*	*Histomonas meleagridis*, *Paenibacillus larvae*, bacteria, parasites	Poultry, Honey bees	[53]
Indolicidin	Cattle (bovine neutrophil peptide)	*Giardia lamblia*	Cattle (calves) or general	[42,55]
Chicken NK-2 (cNK-2)	Chicken (NK-lysin peptide 2)	*Eimeria acervulina* (coccidian parasite)	Poultry (broiler chickens)	[56]
Sophorolipids (SL1–SL4)	Candida bombicola and other yeasts	*Eimeria maxima* and *Clostridium perfringens*	Chicken	[35]
Dermaseptin-SP2	*Agalychnis spurrelli* (frog)	*Plasmodium falciparum*, *Leishmania mexicana*, *T. cruzi*	Livestock parasite models	[57]

**Table 8:** Comparison of AMPs vs. antibiotics’ stability, target specificity, side effects, toxicity, synthetic challenges, future impact, bioavailability.

Criterion	Antibiotics	AMPs
Mechanism of Action	Inhibit specific intracellular processes (e.g., cell wall, protein, or DNA synthesis) through well-defined molecular targets [110].	Disrupt microbial membranes via electrostatic or hydrophobic interactions; some also inhibit intracellular functions after cell penetration [14,111].
Target Specificity	Antibiotics vary in target specificity: some have a broad spectrum of activity, while others target specific bacterial groups. Their selectivity also differs, but they generally act on bacterial structures with minimal toxicity to host cells [107].	Typically broad-spectrum, active against bacteria, fungi, viruses, and parasites, although some, such as bacteriocins, can be narrow spectrum. They are usually selective for microbes due to differences in membrane charge, but excessive hydrophobicity may increase toxicity to host cells [108,110].
Speed of Action	Slower acting; therapeutic effects may take hours to days [14].	Fast-acting; often kill microbes within minutes of exposure [14].
Bioavailability	Generally good oral bioavailability; systemically absorbed and distributed, with tissue residues observed (e.g., salinomycin, enrofloxacin) [9,72].	Limited by poor stability and rapid degradation; mainly topical use [112].
Microbial Resistance	High risk of resistance development via multiple mechanisms, including efflux pumps, target modification, and gene transfer [107].	Lower risk of resistance; some adaptive responses exist, but classical resistance is less common and slower to emerge [113].
Toxicity to Host	Generally low, but some may disrupt microbiota or trigger inflammation [2].	Typically low at therapeutic doses, but toxicity can increase with concentration or structural modifications [108].
Stability	Generally stable; specific properties depend on the compound [114].	Often unstable in biological environments, though stability can be enhanced via self-assembly or chemical modification [108].
Side Effects	May trigger inflammatory responses and gut dysbiosis; bacterial lysis can release endotoxins [2].	Act as immunomodulators; regulate inflammation, recruit immune cells, and support gut and barrier health [111].
Application	Widely used in human and veterinary medicine; also used in livestock for disease control and formerly for growth promotion [2].	Promising alternatives under development; used in medicine, agriculture, aquaculture, and food preservation; clinical use still limited [108].
Synthetic Challenges	Main challenge lies in discovering new effective compounds; clinical translation requires thorough safety and compatibility testing [113].	High synthesis cost and low yield limit large-scale use; biological expression is promising but faces issues like host toxicity and degradation [14].
Future impact	Growing resistance limits effectiveness and raises health risks; calls for new drugs and more responsible use [2].	Promising alternative for resistant infections; broad potential in medicine, agriculture, and animal health [108].

## Data Availability

Not applicable.

## Abbreviations

The following abbreviations are used in this manuscript:

AMP	Antimicrobial Peptide
AFP	Antifungal Peptide
AMR	Antimicrobial Resistance
HDP	Host Defense Peptide
ROS	Reactive Oxygen Species
